# Sequence-Based Analysis of Structural Organization and Composition of the Cultivated Sunflower (*Helianthus annuus* L.) Genome

**DOI:** 10.3390/biology3020295

**Published:** 2014-04-16

**Authors:** Navdeep Gill, Matteo Buti, Nolan Kane, Arnaud Bellec, Nicolas Helmstetter, Hélène Berges, Loren H. Rieseberg

**Affiliations:** 1 Department of Botany and The Biodiversity Research Centre, University of British Columbia, Vancouver V6T 1Z4, BC, Canada; E-Mail: loren.rieseberg@botany.ubc.ca; 2 Applied Rosaceous Genomics Group, Centre for Research and Innovation, Michele all'Adige (TN) P.IVA 020384102, Italy; E-Mail: matteo.buti@fmach.it; 3 Department of Ecology and Evolutionary Biology, University of Colorado, Boulder, CO 80309, USA; E-Mail: nckane@gmail.com; 4 French Plant Genomic Resource Centre, INRA–CNRGV, Chemin de Borde Rouge, CS 52627, 31326 Castanet Tolosan, France; E-Mails: Arnaud.Bellec@toulouse.inra.fr (A.B.); N.Helmstetter@exeter.ac.uk (N.H.); hberges@toulouse.inra.fr (H.B.)

**Keywords:** sunflower, genome, whole genome duplication, transposable elements, Class I LTR-retrotransposons, Class II DNA transposons, transcriptome, expressed sequence tags, expression pattern

## Abstract

Sunflower is an important oilseed crop, as well as a model system for evolutionary studies, but its 3.6 gigabase genome has proven difficult to assemble, in part because of the high repeat content of its genome. Here we report on the sequencing, assembly, and analyses of 96 randomly chosen BACs from sunflower to provide additional information on the repeat content of the sunflower genome, assess how repetitive elements in the sunflower genome are organized relative to genes, and compare the genomic distribution of these repeats to that found in other food crops and model species. We also examine the expression of transposable element-related transcripts in EST databases for sunflower to determine the representation of repeats in the transcriptome and to measure their transcriptional activity. Our data confirm previous reports in suggesting that the sunflower genome is >78% repetitive. Sunflower repeats share very little similarity to other plant repeats such as those of Arabidopsis, rice, maize and wheat; overall 28% of repeats are “novel” to sunflower. The repetitive sequences appear to be randomly distributed within the sequenced BACs. Assuming the 96 BACs are representative of the genome as a whole, then approximately 5.2% of the sunflower genome comprises non TE-related genic sequence, with an average gene density of 18kbp/gene. Expression levels of these transposable elements indicate tissue specificity and differential expression in vegetative and reproductive tissues, suggesting that expressed TEs might contribute to sunflower development. The assembled BACs will also be useful for assessing the quality of several different draft assemblies of the sunflower genome and for annotating the reference sequence.

## 1. Introduction

Cultivated sunflower (*Helianthus annuus* L.) is a globally important oilseed, food, and ornamental crop, ranking 11th among the world’s food crops in terms of area harvested [1]. It is the only major crop to have been domesticated in North America [2,3] and represents the “cornerstone” of the eastern North American domestication hypothesis [4]. Sunflower belongs to the daisy family Compositae, which is one of the largest and most ecologically diverse families of flowering plants [5]. However, genomic characterization of sunflower and other Compositae species has been slow, in part because Compositae crops have very large genomes. A reference genome is not yet available for sunflower, and the organization and structure of the sunflower genome remains poorly understood. This impedes research in sunflower and other Compositae species, and hinders the facile application of molecular approaches to sunflower breeding and improvement.

The sunflower genome is fairly large and complex. It contains between 3.5 and 3.6 billion bases [6], making it roughly 15% larger than the human genome. The majority of the sunflower genome is composed of repetitive sequences, mainly transposable elements [7,8,9,10]. Transposable elements are a ubiquitous feature of eukaryotic genomes and are instrumental in gene regulation [11,12], genome size evolution [13,14], and higher order physical (re)structuring of genomes [15], including chromosomal rearrangements, which may be an important contributor to speciation [16,17].

Comparative analyses reveal that viral and prokaryote genomes are mainly comprised of coding sequence and therefore gene number scales closely with genome size [18]. However, this correlation breaks down in Eukaryotes. While gene number increases gradually with genome size in small Eukaryotic genomes (<100 Mbp), in larger Eukaryotic genomes most genome size variation is a consequence of changes in the abundance of spliceosomal introns and mobile genetic elements [19]. Plant genomes differ from animal genomes of comparable size in having an unusually large number of genes, but less intronic DNA. The expansion of gene number in plants is due partly to gene amplification, for example in rice [20] and whole genome duplication events, for example in maize [21].

Like most flowering plant species, the sunflower genome is a product of several whole genome duplications [22]. These include a basal Compositae paleopolyploidization (40–45 Ma) and a basal Heliantheae paleopolyploidization (26–31 Ma). Polyploidy has significant genomic consequences beyond gene and genome duplication. These include increased rates of karyotypic evolution, as well as rapid changes in the number, expression, and splicing of genes [23,24,25].

Like most other eukaryotes, sunflowers have a predominance of Class I long terminal repeat retrotransposons (LTR-RTs) in their genomes [8,26,27,28,29,30]. These retrotransposons belong to a class of mobile genetic elements that propagate via a mechanism similar to the replication of retroviruses [31], also known as the “copy and paste” mechanism, thereby increasing in copy numbers as they move around in the genome. The distribution, localization and evolution of Ty1-Copia and Ty3-Gypsy families belonging to Class I LTR-RTs have been studied extensively in the genus *Helianthus* [9,32,33,34] and have been proposed to play a role in the evolution of homoploid hybrid species [35]. While these elements have proliferated in three ancient homoploid hybrid species, proliferation in contemporary hybrid populations appears to be rare [36,37,38].

Here we investigate how repetitive elements in the sunflower genome are organized relative to genes and how this arrangement compares to other agriculturally important food crops. Toward this end, we sequenced and assembled 96 randomly chosen Bacterial Artificial Chromosome (BAC) clones. To ensure our results were representative of the genome as a whole, we compared results from the 96 BACs to ~80× coverage of the sunflower genome based on Ilumina whole genome shotgun (WGS) sequencing that was conducted as part of an ongoing genome sequencing effort [8]. We also exploited deep transcriptome sequencing to identify the transcribed portion of the genome and the representation of repeats in the transcriptome. The present paper differs from previous studies [9,10] in the larger number and less biased choice of BACs for sequencing, the much greater depth of WGS available for extrapolating genome-wide patterns, as well as in the characterization of the gene content of the sunflower genome. The information generated represents a key step in the ongoing sunflower genome sequencing project [8], with important implications for sequencing, assembly, annotation, and genetic and physical mapping strategies.

## 2. Experimental

### 2.1. Sequencing and Assembly of the BAC Clones

The BAC library was constructed for the elite cultivated line, HA412-HO, by the French Plant Genome Resource Center [39] by partial digestion of genomic DNA with *Hind*III. Ninety-six BACs were arbitrarily chosen for sequencing with the Illumina GA II sequencing system (Supplementary Table S1).

Paired-end Illumina reads of the 96 BACs were *de novo* assembled with CAP3 [40] and CLC Genomics Workbench [41] using default parameters and the following settings: Length fraction = 0.4, Similarity = 0.9, Non-specific matches = Ignore. Contigs from the two assemblies were scaffolded with S-Space [42]. BWA [43] with default parameters, was used to map the raw reads against the resulting scaffolds, and SAMtools [44] was used for downstream analysis. A custom Perl script was used to determine the average coverage per scaffold. Scaffolds with average coverage less than 100 were eliminated. Vector sequences including pIndigo BAC-5 were identified with BLASTN and removed [45]. Most BAC assemblies were fragmented, most likely due to the highly repetitive nature of the sunflower genome. The assembled BACs have been submitted to GenBank (GenBank accessions: AC254865; AC254997-AC255082; AC255084-AC255092).

### 2.2. Identification and Annotation of the Repetitive Fraction of the Genome

RECON, an open-source software package for *de novo* repeat identification and classification [46], was used to identify repeats in the 96 sunflower BACs. To increase the speed and efficiency of the program, the BLAST output was parsed to discard self-hits, as well as hits with an *e*-value greater than 1 × *e*^−5^. The RECON output was parsed for sequences greater than 50 bp in length that were found at least five times per family. As a complementary approach, *de novo* repetitive sequences were also identified using RepeatScout [47], and the overlap was determined by RepeatMasker version 3.1.9 [48].

BLASTN, BLASTX [49], and TransposonPSI [50] searches against the all-plant repeat database [51] were used to annotate the *de novo* repeats. An *e*-value cut-off of 1 × *e*^−5^ was employed for these searches. Repeats were compiled into a custom repeat database and used for homology-dependent repeat search using RepeatMasker. Custom Perl scripts were used to parse the RepeatMasker results to remove/minimize any overlaps between the different repeat co-ordinates and to calculate the abundance of each repeat in our dataset. The un-annotated novel repeats were used in a cluster analysis using Blastclust [52] at the following settings—L = 0.51 S = 80. Low-complexity repetitive regions and simple sequence repeats (SSRs) were also identified, and their relative abundance and density were determined. The frequencies of different SSR motifs within each di-, tri-, and tetranucleotide repeats were estimated as well. 

#### 2.2.1. Mathematically Derived Repeats

Tallymer [53], a program based on enhanced suffix arrays [54], was used to compute the 20-mer occurrence counts and construct a frequency index of each 20-mer. These frequencies were plotted logarithmically on a genomic scale to distinguish regions of high TE content from low copy regions. Based on the 20-mer frequency distribution, BAC clones were further categorized into low, mid and high repetitive clones.

#### 2.2.2. Class I LTR-Retrotransposons

Class I LTR-retrotransposons were identified using LTR-finder [55] at default parameters. LTRs of each predicted retrotransposon were analyzed with J-dotter [56] and ClustalX [57] to define their boundaries and to eliminate the false hits. The LTR-RTs were annotated based on BLASTN and BLASTX searches against the NCBI non-redundant database at an *e*-value of 1 × *e*^−5^. Clusters of nested repeats were identified by TE Nest [58]. LALIGN version 35.04 February 20, 2010 was used to find non-overlapping local alignments [59].

#### 2.2.3. Coverage of LTR-RTs in the Genome

Whole genome shotgun Illumina reads from a 200bp insert library that provides ~35× coverage of the sunflower genome (GenBank accession: SRX264540) were mapped against the LTR-RTs using BWA at default parameters. BWA output files were manipulated using SAMtools and, using customized Perl scripts, average coverage was calculated for each element.

#### 2.2.4. Estimation of Insertion Age of LTR-RTs and Other TE Families

Insertion time estimates of Class I LTR-RTs were based on the occurrence of nucleotide substitutions between the 5' and 3' LTRs of a LTR-RT. DnaSP5 [60] was used to calculate the number of polymorphic sites for each LTR pair. Insertion age was estimated using the formula T = d/2r, where d is the likelihood divergence estimate for each LTR-RT estimated using the Kimura 2-parameter method [61] and r = 2.0 × 10^−8^ (as calculated by [37]), which assumes that the mutation rate of LTR-RT’s is approximately double the silent site mutation rate for sunflower.

A consensus-based approach was also used to infer the age of TE families [62,63,64]. For each TE family, the number of pairwise nucleotide substitutions to the consensus per TE was determined using DnaSP5 and used to calculate the average number of substitutions relative to the consensus (k). The approximate age of the TE family was estimated using the formula T = kr, where r = 2.0 × 10^−8^ (as above).

### 2.3. Identification and Annotation of the Genic Content of the Genome

AUGUSTUS, an *ab initio* annotation program [65] was used to predict genes in both the repeat-masked and the unmasked datasets (Parameters: Alternative scripts = none, Allowed gene structures = only predict complete genes; Training set = *Arabidopsis thaliana*). The augustus predictions were verified by BLASTN (MegaBlast) and BLASTX (1 × *e*^−15^) against the NCBI *Helianthus* EST database and the NCBI non-redundant protein database, respectively, and also parsed to eliminate the predictions without start and stop codons.

#### Gene Ontology (GO) Annotation

To determine the functional annotation of the predicted genes, and to look for differences between the repeat-masked and unmasked gene prediction datasets, we used both sets of sequences for GO analysis. The two sets of sequences were used as queries to the NCBI non-redundant database using BLASTX (1 × *e*^−35^). The BLAST output in the XML format was imported into BLAST2GO (B2G) for GO analysis by mapping each blast-based high-identity match to an associated GO annotation term [66]. The resulting annotations were converted into the “GO-Slim” format and retrieved for the three GO categories (biological process, molecular function and cell component) with an alpha score of at least 0.6 and an ontology depth level of 3.

### 2.4. Transcriptome Analysis

To identify the transcribed portion of the genome and representation of repeats in the transcriptome, both the repeat-masked and the unmasked datasets were used to screen the EST clusters in all six translated frames using TBLASTX at 1 × *e*^−35^. A total of 477,922 long read (Sanger) EST sequences from four plant species both within Compositae (sunflower—31,605 sequences and lettuce—50,433 sequences [67] and outside Compositae (rice—247,516 sequences and *Arabidopsis*-148,368 sequences, NCBI Taxon ID: 4530 and 3702, respectively) were used for this analysis. To determine the differences in expression patterns of TEs between the transcriptome and the whole genome, average coverage of 256 LTR-RTs was determined (as described previously in Section 2.2.3) for flower and root-stem HA412 RNA-seq libraries (GenBank Accessions: SRX475914; SRX475915) and compared with their coverage in the whole genome.

### 2.5. Phylogenetic Analysis

Reverse transcriptase domains were used to infer the evolutionary history and dynamics of the two major types of LTR-RTs—*Ty1-Copia* and *Ty3-Gypsy*. RT-domains homologous to those of the sunflower copia and gypsy elements were identified from rice [68], *Arabidopsis* [69], maize [70] and *Selaginella* [71] by BLASTX (1 × *e*^−5^). Multiple alignments were performed by MUSCLE [72] and manually edited in Jalview [73]. The evolutionary distances were computed using the Poisson correction method [74] and are in the units of the number of amino acid substitutions per site. The analysis involved 75 *Ty1-Copia* and 110 *Ty3-Gypsy* amino acid sequences. An unrooted 1000 bootstrap Neighbor-Joining tree [75] was constructed in MEGA5 [76].

## 3. Results

An arbitrarily chosen set of 96 BACs consisting of 955 scaffolds and amounting to 14,058,762 bp (0.4% of the 3600 Mbp genome) was analyzed to characterize the repeat and genic content of the sunflower genome. A combination of *de novo* and homology-based methods was used to identity and annotate repetitive elements.

### 3.1. Abundance, Distribution, Amplification and Divergence of Repetitive Elements in the Sunflower Genome

#### 3.1.1. Abundance

We created a sunflower custom repeat library through *de novo* identification (RECON) and annotation (using TransposonPSI and BLAST searches) of repetitive sequences. A total of 6956 repetitive elements belonging to 682 repeat families and ranging from 51 bp to 13,914 bp were identified (available as Supplementary Material). Copy number distribution of these families indicates a preferential amplification of only a few repeat families in the genome (Supplementary Figure S1), with 44 (6% of the total) and 127 (19% of the total) families accounting for 50% of the entire repetitive content of the genome in terms of base pair coverage and copy number coverage, respectively. These families, which likely include centromeric repeats or centromere-associated sequences, represent candidates for future *in-situ* experiments to investigate their physical location in the genome.

**Figure 1:**
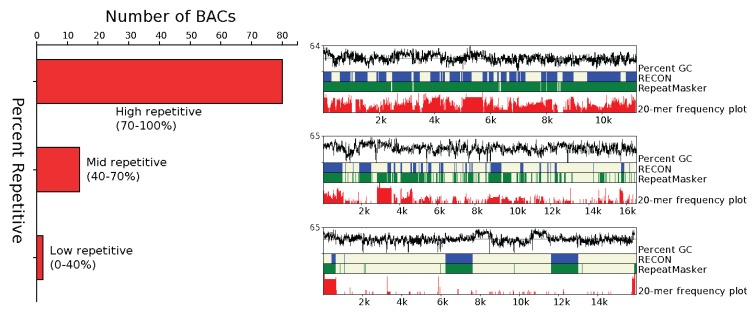
Percent of total BAC clones classified as low, mid and high repetitive based on the frequencies of the overlapping 20-mers for each clone. The right hand side panel is an illustration of each type of BAC clone (low, mid and high repetitive) with tracks showing the percent GC, RECON [46] and RepeatMasker [48] annotations along with the frequency of 20-mers [53] for each clone is shown. The repeat-rich and repeat-poor regions, as shown by the 20-mer frequency plot by Tallymer and supported by the RECON and RepeatMasker annotations can be differentiated easily.

To estimate, characterize and classify the repetitive content of the sunflower genome further, the following analytical approaches were employed: LTR-Finder, RepeatMasker and Tallymer. The majority (83%) of the BACs were categorized as highly repetitive (70%–100% repetitive), 15 percent as mid repetitive (40%–70% repetitive) and two percent as low repetitive (0%–40% repetitive) as shown in Figure 1.

Assuming a representative dataset, approximately 78% of the genome is estimated to be repetitive with a repeat density of 433 bp/repeat in the repeat-dense potentially heterochromatic and 12,279 bp/repeat in the repeat-poor (containing single or low copy sequences) potentially euchromatic regions of the genome, and an average density of 815 bp/repeat (Table 1). The transposable element landscape of the sunflower genome is dominated by the presence of Class I LTR-retrotransposons that comprise ~67% of the genome and consist of two super-families—*Ty1Copia* and *Ty3Gypsy*. This estimate does not include the solo-LTRs, which given the fragmentary nature of the data, were difficult to confirm. Class II DNA transposons including both Miniature Inverted Transposable Elements (MITEs) and non-MITE DNA transposons such as Cacta, En/Spm, Mariner, Mutator and Snoopy comprised a mere 0.4% of the genome. Ribosomal repeats (45S rDNA and 5S rDNA), centromeric satellite repeats and telomeric-associated sequences collectively comprise 0.5% of the genome.

Simple Sequence Repeats (SSRs) and low complexity regions account for 1.1% of the genome. Among the SSRs, the tri-nucleotide motifs were the most abundant in the genome with 7728 repeats units on average (Supplementary Table S2). Of the top 10 SSR motifs present in the genome in order of their abundance, eight are tri-nucleotides with TGG/CCA as the most frequent tri-nucleotide motif in the genome (Supplementary Figure S2). Low complexity regions in other plants are typically comprised of A/T, AT, GA/TC, CT/AG, GC, G/C, polypurine and polypyrimidine-rich regions but in the sunflower genome, they are predominantly AT-rich (~82% of the total; Supplementary Figure S3).

**Table 1:** Repeat composition of the sunflower genome as determined by RECON [46], RepeatScout [47] and RepeatMasker [48].

REPEAT CLASS	REPEAT TYPE	TOTAL NUCLEOTIDES IN THE BAC DATASET (bp)	TOTAL NUCLEOTIDES IN THE GENOME (bp)	^$^ PERCENT OF THE GENOME	PERCENT OF TOTAL REPETITIVE
Classs I Retrotransposons					
	Ty1-Copia	2,014,560	515,864,483,658	14.33	16.28
	Ty3-Gypsy	2,633,637	674,390,333,943	18.73	21.28
	LINEs, SINEs	14,316	3,665,870,437	0.10	0.12
	* Unclassified	2,307,645	590,914,192,871	16.41	18.65
	** Novel	2,463,529	630,831,107,319	17.52	19.91
	**Subtotal**	**9,433,687**	**2,415,665,988,229**	**67.10**	**76.24**
Class II DNA Transposons					
	^#^ Non-MITEs	19,451	4,980,779,958	0.14	0.16
	MITEs	3,442	881,386,284	0.02	0.03
	Unclassified	32,398	8,296,093,212	0.23	0.26
	**Subtotal**	**55,291**	**14,158,259,454**	**0.39**	**0.45**
Ribosomal DNA	5S, 45S	34,127	8,738,834,899	0.24	0.28
Centromeric Repeats		32,353	8,284,570,149	0.23	0.26
Telomeric Repeats		1,281	328,023,193	0.01	0.01
SSRs and Low Complexity		160,912	41,204,424,685	1.14	1.30
Unclassified Repeats		1,194,287	305,818,762,705	8.49	9.65
** Other Novel Repeats		1,462,325	374,454,735,061	10.40	11.82
**Total Repetitive**		**12,374,263**	**3,168,653,598,375**	**^$$^ 88.02**	

^$^ Percentages are based on a genome size of 3.6 Gb; ^$$^ Overestimation of total repeat content (88% *vs*. 78%) is due to overlapping repeat boundaries and nested TEs; * Unclassified repeat--present in the all plant repeat database but classification is unknown; ** Novel repeat—no similarity to the known repeats, could be diverged or mutated beyond recognition or exclusive to the sunflower genome; ^#^ Non-MITE DNA TEs include Cacta, En/Spm, Mariner, Mutator and Snoopy.

Approximately 28% of the repetitive elements identified in sunflower are novel, of which at least 18% are Class I LTR-RTs as was determined by comparing the novel repeats with the output of LTR-finder. The novel repeats, in this case are sequences that are identified as being repetitive by *de novo* repeat finding algorithms, but show no homology to either the TIGR all plant repeat database or to any other sequence in the NCBI non-redundant databases. Such sequences could either be truncated, diverged or mutated beyond recognition, making it hard, almost impossible, for detection by homology based methods, or they could be sequences that are exclusive to the sunflower genome, verification of which is beyond the scope of this manuscript. We, however, performed a blastclust based clustering analysis of these 6286 novel sequences (Table 2) and obtained 1335 clusters with the largest cluster containing as many as 139 sequences (2.2% of the total). This indicates the presence of sub-groups of novel repeats based on sequence homology of at least 80% over at least 51% of their length.

There is a considerable variation in the transposable element composition between sunflower and other model monocot and dicot plant species for instance, *Arabidopsis*, rice and maize (Supplementary Figure S4). Class I LTR-RTs in small-sized genomes such as *Arabidopsis* and rice comprise approximately 3 and 18 percent of the genome, respectively, which is fairly small as compared to the Class I LTR-RT content in the relatively larger genomes such as maize and sunflower (55 and 67 percent, respectively). The amount of LTR-RTs in each of these genomes is perfectly correlated with the genome size of the species (r = 1.0). Also, an overlap of 15, 16, 14 and one percent was observed between the sunflower *de novo* repeats and the *Arabidopsis*, rice, maize and wheat repeats, respectively suggesting that the sunflower repeats share little similarity to other model monocot and dicot plant repeats available to date.

**Table 2:** Blastclust (L = 0.51 S = 80) based clustering analysis of the “Novel” Sunflower repeats that did not show any significant hits to the previously annotated repeats or to the TIGR all plant repeat database.

Total number of sequences	6286
Number of sequences not clustered	97
Number of clusters	1335
Clusters with >5 sequences	346
Clusters with >20 sequences	27
Clusters with >50 sequences	8
Clusters with >100 sequences	4
Number of sequences in the largest cluster	139

#### 3.1.2. Distribution

To determine the distribution and organization of repetitive sequences, we identified 35 BACs with a minimum contiguous sequence of 50 kb. These BACs were divided into 5 kb bins, and the organization of repetitive sequences in each of those bins was determined (Supplementary Figure S5). The non-parametric runs test for randomness [77] was used to determine whether the distribution of repetitive sequences across the scaffold length is random. Regions defined as repetitive in our analysis (as described in the previous section) were denoted by 1, putative euchromatic regions by 0, and their distribution was analyzed by the standard one-sample runs test. At a significance level of *p* < 0.05, only three BACs—namely BAC 32 († *p* = 0.007), 83 († *p* = 0.015) and 84 († *p* = 0.044) follow a nonrandom distribution of repetitive sequences, while the rest show a random distribution (Supplementary Figure S6).

We also observed the presence of multiple transposable elements found inserted within each other, often referred to as nested TEs (data not shown). The age of the insertion as determined by the sequence divergence between the LTRs of a retrotransposon [78], as well as the number of LTR-RTs that formed the nested structure, varied among the different insertions. Nested TEs are a common occurrence in highly repetitive genomes such as maize [79], where a majority of the TEs are found inserted into the sequence of an existing element, creating complex structures that are difficult to resolve and compare. Such clustering results in the generation of large methylated and heterochromatic blocks [80] and may serve as a genome-defense mechanism to avert the lethal effects of TE insertions into or near genes.

#### 3.1.3. Amplification and Divergence

Given that the TE families arise via amplification from a few or a single TE, also known as the ancestral element(s) for that family, the consensus sequence is a fairly accurate approximation of the ancestral TE sequence [63,81]. We used TE consensus [62,63] to infer the age of 233 TE families with at least 10 members/family and a minimum consensus length of 100 bp. This approach can be applied to all types of TEs and is not limited to LTR-RTs alone, where the insertion age is inferred by the number of polymorphic sites between the LTR pairs. The average divergence/TE family varies from 0% to 34%, with 85% of the TE families (199 out of 233 total) falling in the range of 10%–30% (Supplementary Figure S7). This indicates the presence of highly divergent TEs in the sunflower genome, and only two families could be identified that were 0% diverged from their consensus. 

**Figure 2:**
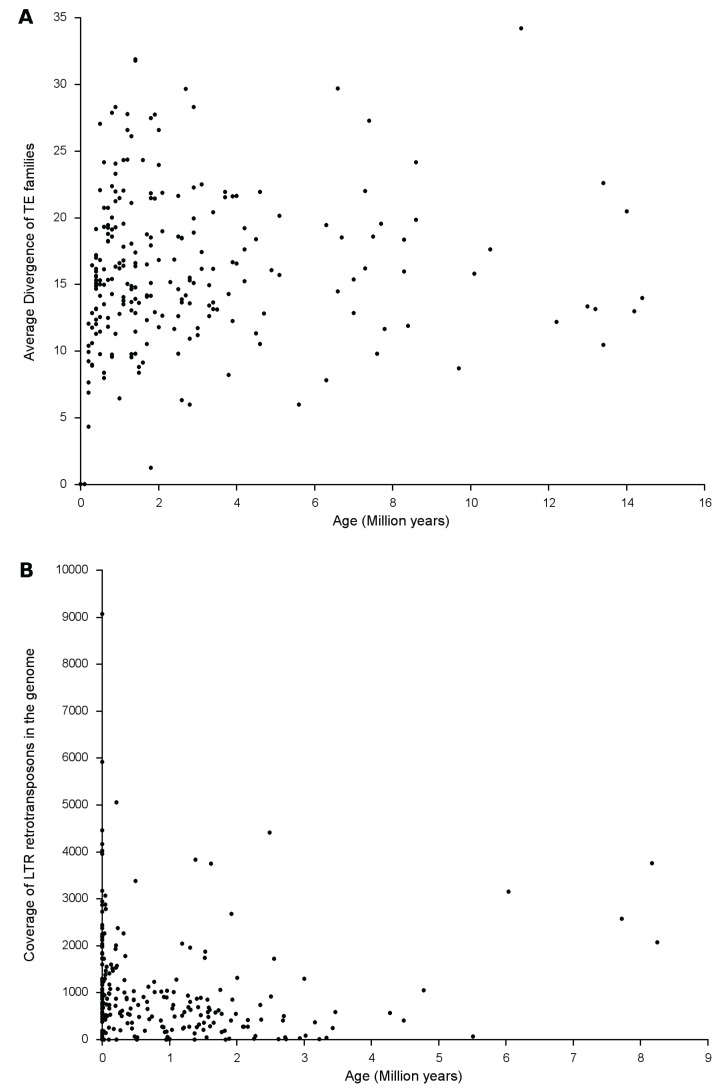
(**a**) The average divergence of Transposable Element (TE) families. The age of the TE families was estimated using the TE consensus approach [62,63,64] using the formula T = kr, where r = 2.0 × 10^−8^ [37] (**b**) Amplification of LTR-retrotransposons in the sunflower genome as a function of its age. Insertion time estimates of Class I LTR-RTs were calculated using the formula T = d/2r, where d is the likelihood divergence estimate for each LTR-RT estimated using the Kimura 2-parameter method [61] and r = 2.0 × 10^−8^ [37].

Insertion ages of the TE families ranged from 0 MY to 14.4 MY (Figure 2a). Approximately, one-third of the TE families (77 out of 233) had insertion ages either equal to or greater than the average insertion age of 2.7MY. The age of a given TE family, however, depends on the number of average variable sites to the family consensus (is perfectly correlated with a correlation coefficient of 1.0). For a given TE family, the average percent divergence to the consensus directly correlates to the time elapsed since most of the insertions occurred, but this not does necessarily hold true when comparing the average percent divergences across different families.

We also calculated the insertion ages of 256 LTR-RTs based on the formula T = d/2r, where d is the likelihood divergence estimate for each LTR-RT estimated using the Kimura 2-parameter method and r = 2.0 × 10^‑8^. Coverage estimates of the 256 LTR-RT families across the whole genome imply that the insertion age of an element does not necessarily correlate (correlation coefficient r = 0) with its amplification (Figure 2b) suggesting a genome-wide defense mechanism that sets a limit to the amplification and proliferation of these particular types of TEs.

#### 3.1.4. Phylogenetic Analysis

**Figure 3:**
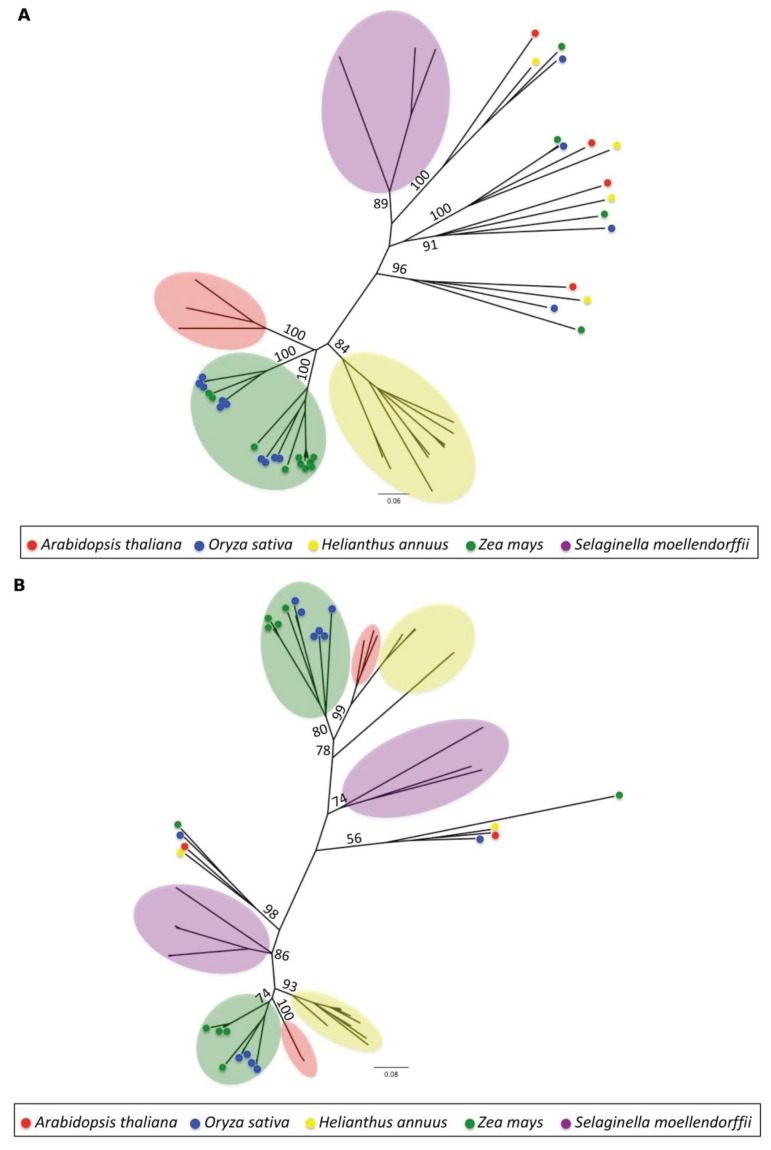
1000 bootstrap Neighbor-Joining tree of (**a**) *Ty1-Copia* and (**b**) *Ty3-Gypsy* elements from different species. The evolutionary history was inferred using the Neighbor-Joining method [75]. The optimal tree with the sum of branch length = 8.7 is shown. The trees are drawn to scale, with branch lengths in the same units as those of the evolutionary distances used to infer the phylogenetic tree. The evolutionary distances were computed using the Poisson correction method [74] and are in the units of the number of amino acid substitutions per site. The analysis involved 75 *Ty1-Copia* and 110 *Ty3-Gypsy* sequences. All ambiguous positions were removed for each sequence pair. There were a total of 250 positions in the final dataset. Evolutionary analyses were conducted in MEGA5 [76].

To establish cross-species phylogenetic relationships for the two LTR-RT super-families that comprise roughly 70% of the sunflower genome, we used the amino acid sequences of the reverse transcriptase domain of *Ty1-Copia* and *Ty3-Gypsy* LTR-RTs from sunflower *Arabidopsis*, rice, maize and *Selaginella* to build neighbor-joining trees (Figure 3a,b). Reverse transcriptase domains of four *Ty1-Copia* families (16 out of 75 *Ty1-Copia* LTR-RTs) share similarity with, and pair closer to *Selaginella* than to other sunflower sequences. These four types of sunflower *Ty1-Copia* LTR-RTs therefore have reverse transcriptase domains that predate the divergence of flowering and non-flowering plants*.* The remaining TE families appear to have arisen after the divergence of the sunflower lineage from the other four taxa included in this comparison. Similarly, only two *Ty3-Gypsy* families failed to cluster with other sunflower sequences. However, in both the cases, the sunflower reverse transcriptases are more closely related to *Arabidopsis* than they are to rice, maize or *Selaginella*.

While most sunflower LTR-RTs cluster most closely with other sunflower sequences, a different pattern has been reported for rice and maize, in which LTR-RT families are shown to be frequently more closely related to each other than to families within the same species [82]. Presumably this reflects the more recent divergence between rice and maize than between sunflower and *Arabidopsis*.

### 3.2. Genic Content of the Sunflower Genome

Using the annotation program, AUGUSTUS [65], 2467 and 758 genes were predicted in the unmasked and the repeat-masked datasets, respectively. After manual verification and elimination of predictions without the start and stop codons, we obtained a final predicted gene count of 2321 (unmasked) and 643 (repeat-masked). These predictions were verified by BLASTN (using megablast) against the *Helianthus* EST database and BLASTX against the nr database at an *e*-value threshold of 1 × *e*^−15^. A summary of the genes and genic features are shown (Table 3). Gene densities in the gene-rich (maximum gene density) and gene poor (minimum gene density) regions of the genome were computed by plotting the actual distribution of the genes in OmniMapFree [83]. A greater than 3-fold difference in the average gene density between the repeat masked and the unmasked datasets was observed. Gene families are abundant in both the datasets with as many as >100 genes/family in the unmasked dataset (Supplementary Figure S8). Differences in the distribution of gene family size between the unmasked and repeat-masked sets also indicate the presence of large TE-related gene families in the sunflower genome.

**Table 3:** AUGUSTUS [65] predicted genes and their genic features in the sunflower genome. Both the repeat-masked and the unmasked datasets were used with the following parameters: Alternative scripts = none, Allowed gene structures = only predict complete genes; Training set = *Arabidopsis thaliana*.

BAC Statistics	Unmasked	Repeat-masked
Length (Mb)	14.1	3.4
GC content (%)	39	36.8
Number of predicted genes	2,321	643
BlastN against the Helianthus EST db [proportion of total]	816 [0.4]	209 [0.3]
BlastX against NCBI nr db [proportion of total]	979 [0.4]	304 [0.5]
BlastN and BlastX [proportion of total]	551 [0.2]	160 [0.2]
Average gene size (kb)	3.6	3.0
Gene GC content (%)	41.9	40.0
Minimum gene density (bp/gene)	1,011	4,644
Maximum gene density (bp/gene)	8,042	215,568
Average exon size (bp)	507.6	360.1
Exon size/gene (bp)	640	483.8
Number of exons/gene	4.7	4.1
Exon GC content (%)	44.3	42.7
Average intron size (bp)	339.7	487.1
Intron size/gene (bp)	390.9	618.9
Number of introns/gene	3.7	3.1
Intron GC content (%)	38.5	35.2

**Figure 4:**
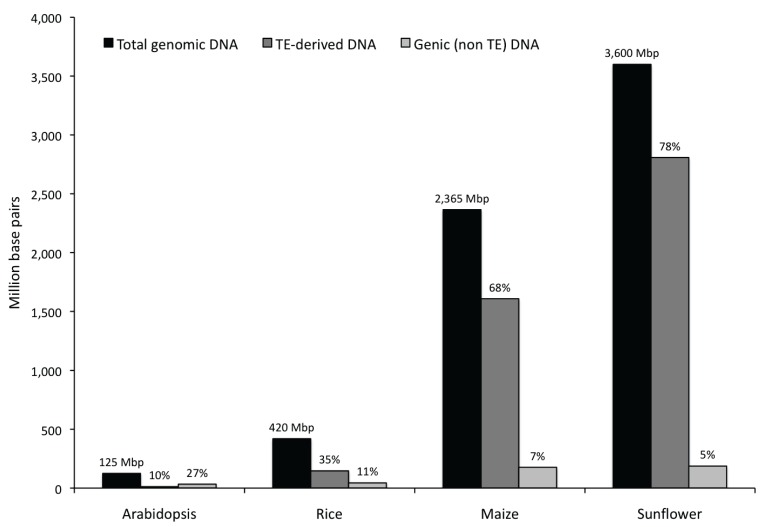
Comparison of the genome size, TE-derived and genic DNA (non TE-derived) among *Arabidopsis*, Rice, Maize and Sunflower [26,27,28].

Overall, 187 Mbp of the 3600 Mbp sunflower genome is estimated to be genic (~5% of the genome). In *Arabidopsis*, rice and maize, 33 Mbp, 44 Mbp and 177 Mbp, respectively, correspond to non-TE related genes in the genome (Figure 4). As the number of genes is more or less the same across different species [26,27,28,29], these differences probably arise from expansions in intron size or number as the genome size increases.

#### Gene Ontology Annotation of the Predicted Genes

**Figure 5:**
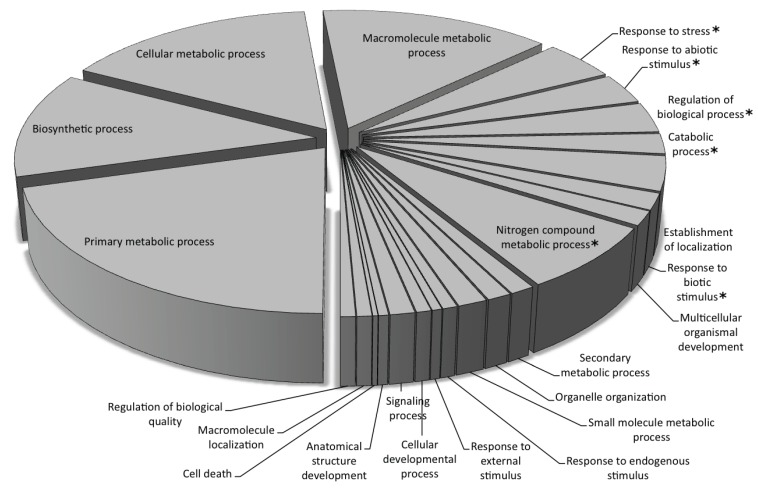
Gene Ontology (GO) annotations of the gene predictions from the repeat-masked dataset in the “Biological process” category using BLAST2GO (B2G) [66] using an alpha score of at least 0.6 and an ontology depth level of 3.

Predicted genes from the repeat-masked and the unmasked datasets were provisionally annotated through BLASTX searches against the NCBI non-redundant database and functionally classified using Blast2GO. Of the 2321 total genes in the unmasked dataset, 1475 had blast hits, of which 1405 were mapped to gene ontology (GO) terms and only 460 (~20% of the total) were annotated. Similarly for the repeat-masked dataset, 366 genes out of 643 had blast hits, 345 were assigned to GO categories and 245 (~38% of the total) were annotated. Based on the association with gene ontology terms, 70% and 93% of the total GO categories for the biological process and molecular function, respectively, were comprised of housekeeping genes (Figure 5; Supplementary Figure S9). While the largest GO categories were similar in the repeat-masked and unmasked datasets, there were significant differences (*p* < 0.01) in the number of annotations for several GO categories (Supplementary Table S3). As expected, the repeat-masked set had significantly fewer annotated genes associated with the integration, multiplication and transposition of transposable elements. Interestingly, GO annotations associated with response to stress, biotic and abiotic stimuli, and endogenous and external stimuli were significantly higher in the repeat-masked dataset, suggesting a role for transposable elements in the regulation of stress-related genes. The higher proportion of GO annotations in the repeat-masked dataset can be attributed to TE or TE fragment insertions into the promoters, introns and/or UTRs of the associated genes, which, when masked, result in an accurate gene prediction and thus increase the fraction of GO terms for those genes. Beyond establishing the role of TEs in gene regulation and genome organization, this analysis demonstrates the importance of repeat identification for better gene identification and annotation, as a part of the ongoing sunflower genome sequencing project [8].

* denotes significant difference between the masked and unmasked datasets at 99% confidence interval.

### 3.3. Transcriptional Activity of the Repetitive Elements in the Sunflower Genome

**Figure 6:**
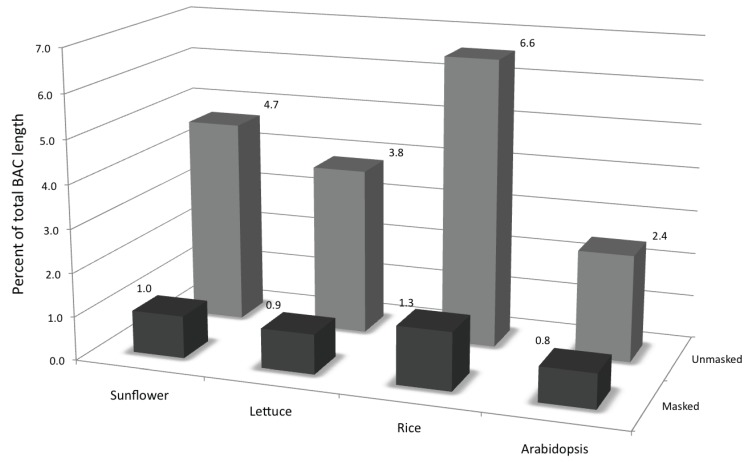
Transcribed portion of the genome as determined by TBLASTX searches of both the repeat-masked and the unmasked datasets against the ESTs of four plant species, two from within Compositae (sunflower and lettuce) and two outside Compositae (rice and *Arabidopsis*).

To identify the transcribed portion of the genome and presence of TE-related transcripts in the sunflower transcriptome, both the repeat-masked and the unmasked datasets were screened against the ESTs of four plant species, two from within Compositae (sunflower and lettuce) and two outside Compositae (rice and *Arabidopsis*). Higher percentages of hits in the unmasked dataset compared to the repeat-masked dataset (Figure 6) indicate the representation of TE-related transcripts in the EST databases. Similar results were observed in all the four species indicating a conserved set of such transcribed TEs, also previously reported in maize [21]. Overall, 2.6% of sunflower ESTs show homology to transposable elements from our custom repeat library, also indicative of the presence of transcripts from transposon-related genes. 

**Figure 7:**
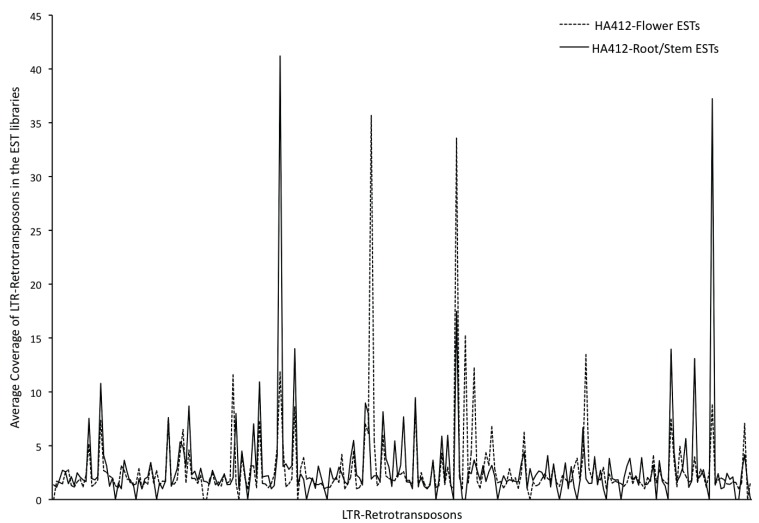
Differential expression of LTR-retrotransposons in the vegetative and reproductive tissues as determined by the average coverage of LTR-RTs in the HA412 flower and root-stem RNA-seq libraries.

As a measure of transcriptional activity, and to determine the differences in expression patterns of TEs between the transcriptome and the whole genome, average genomic coverage of 256 retrotransposons was compared with the flower and root-stem EST libraries (as described in the Experimental). We observed a weak correlation between the coverage of these TEs in the genome and in the transcriptome (r = 0.16), implying that the transcriptional activity of the TEs is not contingent upon their copy numbers in the genome. The expression levels of TEs tested in this study often differed between vegetative and floral tissues (Figure 7; Supplementary Table S4). Nine out of 256 TEs show zero expression in both the root-stem and flower EST libraries, possibly due to the presence of mutated copies in the genome. Seven TEs are exclusively expressed in roots/stems, while 21 are exclusively expressed in flowers. Different patterns of transposon element distribution, amplification and expression in the vegetative and flowering tissues suggest that specific transposable elements may play a role in sunflower development, as well as in the regulation of genes specific to these tissues and/or development stages.

## 4. Discussion

Sunflower is one of the world’s most widely grown crops, but its 3.6 gigabase genome has been proven difficult to assemble, apparently because of the high number and rapid turnover of LTR retrotransposons [9]. We here provide a global view of the organization of the sunflower genome, based on what appears to be a representative subset of the genome, although a more detailed analysis will follow from the ongoing sunflower sequencing project [8]. This study shows for the first time how two main genomic components—genes and repetitive sequences—are distributed and arranged on a genomic scale in the sunflower genome. Our results confirm previous reports that TEs are abundant in the sunflower genome. At least 78% of the genome is repetitive and consists primarily of the Class I LTR-retrotransposons with an average age of 2.7 MY. Our results are comparable to those obtained from the random sampling of genomic data [8,9,10], suggesting that our dataset is indeed representative of the genome as a whole. 

We report a custom sunflower repeat database that can be used as “reference repeats” for Compositae generally and *Helianthus* specifically. Of a total of 6956 repetitive elements belonging to 682 repeat families, our results indicate that preferential amplification of only a few repeat families in the genome (6% and 19% of the total families in terms of bp coverage and copy number coverage, respectively) account for 50% of the entire repetitive content of the genome. Besides polyploidization, differential amplification rates of Class I LTR-RTs is the primary cause for genome size variation among different plant species [84]. The explosive proliferation of Class I LTR-RT families subsequent to speciation, also previously reported in other plant genera such as *Oryza* [14,85], *Zea* [86], and *Gossypium* [87], coupled with their rapid rate of divergence compared to the gene sequences [37,88], make them the major determinants of genome (size) evolution.

Using the non-parametric runs test for randomness [77,89], we observe that with the exception of three BACs, the sunflower repeats appear to be distributed randomly within and between sequence scaffolds. The TEs in the three outlier BACs show greater clustering/organization than expected by chance. Distinct TE clusters are usually marked by increased rates of gene duplications and higher sequence diversity of genes associated with the clusters [90]. We also observe nested TE structures in our analysis. Such structures are known to be formed by the preferential insertion of an LTR-retrotransposon into pre-existing retrotransposons, creating large heterochromatic blocks [79,80]. Other than their potential role in centromere formation [91], and a negative influence on genome expansion [92], the cause and evolutionary significance of such structures largely remains unknown. 

We also observe a high proportion of unclassified repeats possibly due to (1) fragmentary data structure; (2) highly diverged repeats resulting in lack of existing annotation; and (3) “novel” repeats specific to sunflower. It is highly likely that a high proportion of these unclassified and ‘novel’ repeats are LTR-retrotransposons, but it is beyond the scope of this study to test this hypothesis. Such ‘novel’ TEs have potential as species-specific markers for tracking introgression, species identification and phylogenetic analyses.

Gene predictions in the unmasked and repeat-masked sequence suggest that roughly 5.2% of the genome is protein coding. We observe an excess of genes associated with the integration, multiplication and transposition of transposable elements in the unmasked sequence; and stress responsive genes in the repeat-masked dataset, emphasising the potential regulatory roles of TEs. Transcripts of TE related genes were found in the sunflower transcriptome, even though their transcriptional activity did not depend upon their copy numbers in the genome. Such a pattern has also been reported in maize where the rare retrotransposons in the genome are more abundant at the transcript level [82]. These results suggest distinct roles for TEs based on their state of activity and/or amplification in the genome. Differential TE expression patterns in the flowering and vegetative tissues indicate tissue specificity of TEs and possibly a role in sunflower development.

Barbara McClintock’s discovery of TEs [93] was a landmark scientific breakthrough, as was her perception of the transposition mechanism as a genome’s cognitive response to stress [94]. In her view, the genome is “a highly sensitive organ of the cell that monitors genomic activities and corrects common errors, senses unusual and unexpected events and responds to them, often by restructuring the genome” [95]. TE modulated changes to the genome such as insertions, deletions, duplications and translocations have been extensively studied in crops such as *Arabidopsis*, maize, rice, tomato *etc.* [11,13,96,97]. These genome alterations seem likely to contribute to reproductive isolation and speciation, although a direct link has only rarely been made [98]. Moreover, selective proliferation, repression and derepression of specific TEs in a genome has the potential to generate genetic and phenotypic diversity upon which natural selection can act. 

We show that repression of TEs is dependent on their age and copy number in the genome. Old and degenerated copies tend to stop multiplying due to reasons that can either limit their mobility or result in loss of autonomy; for example, accumulation of mutations or deletions in the reverse transcriptase domain of Class I LTR-RTs and/or other proteins can limit transposition. Genomic DNA loss through unequal and illegitimate recombination, on the other hand maintains a genomic balance by counteracting the genomic expansion caused by the Class I LTR-retrotransposons [13,97]. This way genomes do not have a “one-way ticket to genomic obesity” [99] and an “increase-decrease model” [100] is operational to keep the TE copy numbers in check.

## 5. Conclusions

Through detailed sequence analyses of a representative set of 96 Bacterial Artificial Chromosome (BAC) clones, we provide the first report on the overall structural organization as well as sequence composition of the sunflower genome. The assembled BACs will also be useful for assessing the quality of several different draft assemblies of the sunflower genome, and the repeat database reported here will aid in annotation of the sunflower reference genome. Research is ongoing to further characterize the sunflower genome. As more genomic information accumulates, we hope to address some unresolved questions including the (1) origin (e.g., horizontal transfer), evolution, and function of TEs, as well as their fate following polyploidization; (2) impact of TE location and genomic organization on their proliferation and regulation; and (3) the role of transposable elements in gene regulation. On a longer-term basis, we wish to explore how TEs influence the development of reproductive isolating barriers, both directly through the evolution of hybrid incompatibilities and indirectly by facilitating the origin and establishment of chromosomal rearrangements.

## Supplementary Files

Supplementary File 1 Supplementary Materials (ZIP, 2908 KB)

## Supplementary Materials

**Sequence File**—Sunflower repeat library in fasta format.

**Figure S1.** Copy Number Distribution of the Sunflower Repeat Families as identified by RECON [46].

**Figure S2.** Top 10 Simple Sequence Repeat (SSR) motifs arranged in order of their abundance in the sunflower genome.

**Figure S3.** Different types of Low Complexity (LC) sequences identified in the sunflower genome expressed as percentage of total Low Complexity region.

**Figure S4.** Variation in Transposable Element composition in *Arabidopsis*, Rice, Maize and Sunflower.

**Figure S5.** Organization of Repetitive Sequences in the BACs.

**Figure S6.** Runs non-parametric test [77] for randomness to determine the random *versus* non random distribution of repetitive sequences.

**Figure S7.** The average divergence of 233 Transposable Element (TE) families calculated using the TE consensus approach [62,63,64].

**Figure S8.** Estimation of the number and size of gene families based on the gene predictions by AUGUSTUS [65].

**Figure S9.** Gene Ontology (GO) annotations of the gene predictions from the repeat-masked dataset in the “Molecular Function” category using BLAST2GO (B2G) [66] using an alpha score of at least 0.6 and an ontology depth level of 3.

**Table S1.** Assembly Statistics of the 96 BACs used in the analysis.

**Table S2.** Simple Sequence Repeats (SSRs) in the sunflower genome.

**Table S3.** Differences in GO annotations between the unmasked and repeat-masked datasets and Fisher’s Exact Test of Significance.

**Table S4.** LTR-Retrotransposon expression in a. Genome *vs.* the Transcriptome and b. Tissue specific expression of LTR-Retrotransposons.
