# Supplementary material for: Sequence-Based Analysis of Structural Organization and Composition of the Cultivated Sunflower (Helianthus annuus L.) Genome
Source: Biology (Basel). 2014 Apr 16;3(2):295–319. doi: 10.3390/biology3020295 (PMC4085609; doi:10.3390/biology3020295)
Supplement: Supplementary File 1 — Supplementary Materials (ZIP, 2908 KB) [file biology-03-00295-s001.zip › biology-44239-supplementary-final/biology-44239-Supplementary-Figures-final.docx]

*Biology* **2014**, *3*, S1-S6

**OPEN ACCESS**

***biology***

**ISSN 2079-7737**

www.mdpi.com/journal/biology

Supplementary Materials

Sequence-Based Analysis of Structural Organization
and Composition of the Cultivated Sunflower
(*Helianthus annuus* L.) Genome

Navdeep Gill ^1,^*, Matteo Buti ^2^, Nolan Kane ^3^, Arnaud Bellec ^4^, Nicolas Helmstetter ^4^,
Hélène Berges ^4^ and Loren H. Rieseberg ^1^

^1^ Department of Botany and The Biodiversity Research Centre, University of British Columbia, Vancouver V6T 1Z4, BC, Canada; E-Mail: loren.rieseberg@botany.ubc.ca

^2^ Applied Rosaceous Genomics Group, Centre for Research and Innovation, Michele all'Adige (TN) P.IVA 020384102, Italy; E-Mail: [matteo.buti@fmach.it](mailto:matteo.buti@fmach.it)

^3^ Department of Ecology and Evolutionary Biology, University of Colorado, Boulder, CO 80309, USA; E-Mail: nckane@gmail.com

^4^ French Plant Genomic Resource Centre, INRA–CNRGV, Chemin de Borde Rouge, CS 52627, 31326 Castanet Tolosan, France; E-Mails: Arnaud.Bellec@toulouse.inra.fr (A.B.); N.Helmstetter@exeter.ac.uk (N.H.); hberges@toulouse.inra.fr (H.B.)

***** Author to whom correspondence should be addressed; E-Mail: [navdeep.gill@botany.ubc.ca](mailto:loren.rieseberg@botany.ubc.ca);
Tel.: +1-604-827-3535; Fax: +1-604-822-6089.

Received: 30 October 2013; in revised form: 16 March 2014 / Accepted: 25 March 2014 /
Published: 17 April 2014

**Figure S1.** Copy Number Distribution of the Sunflower Repeat Families as identified by RECON [1].


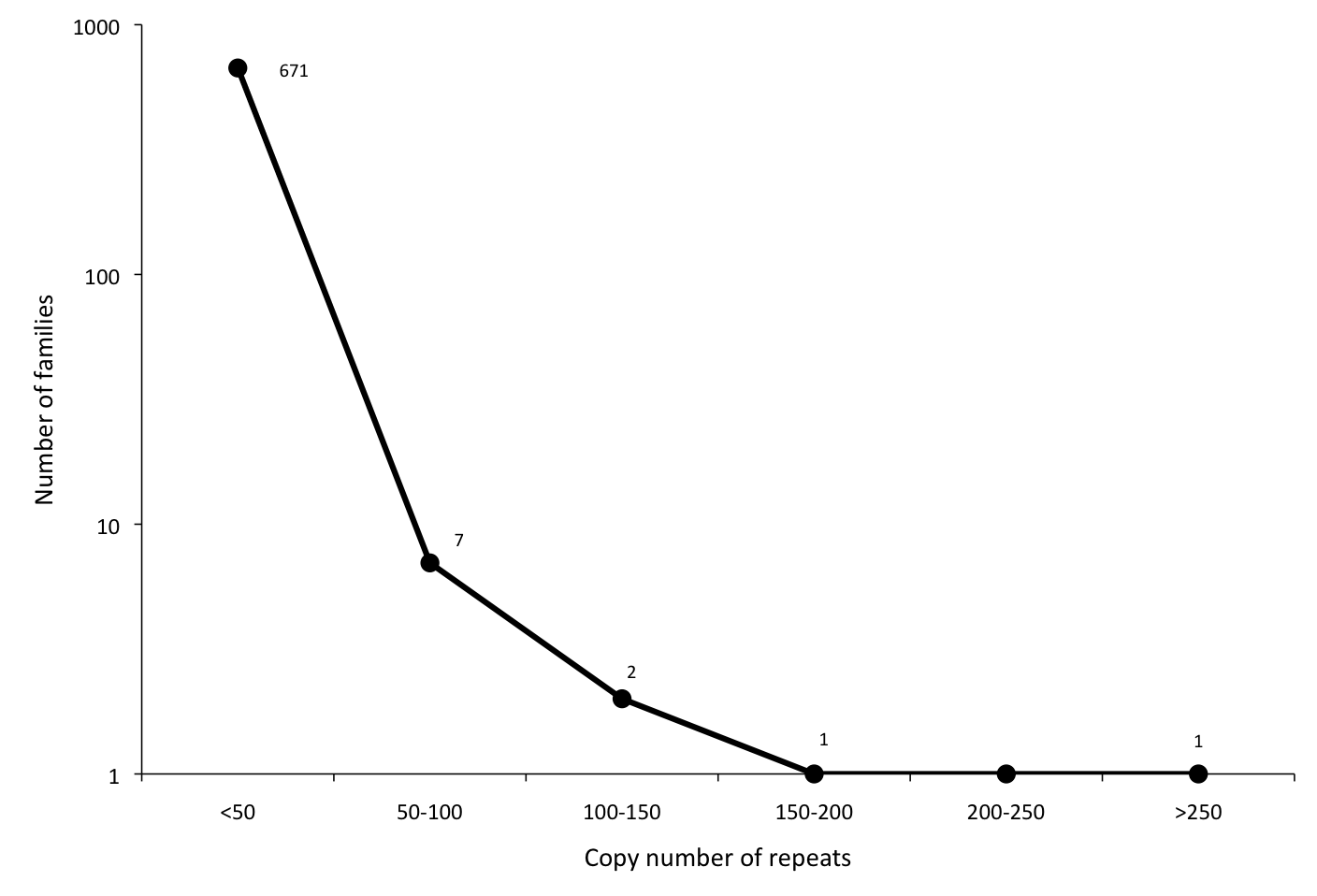


**Figure S2.** Top 10 Simple Sequence Repeat (SSR) motifs arranged in order of their abundance in the sunflower genome.


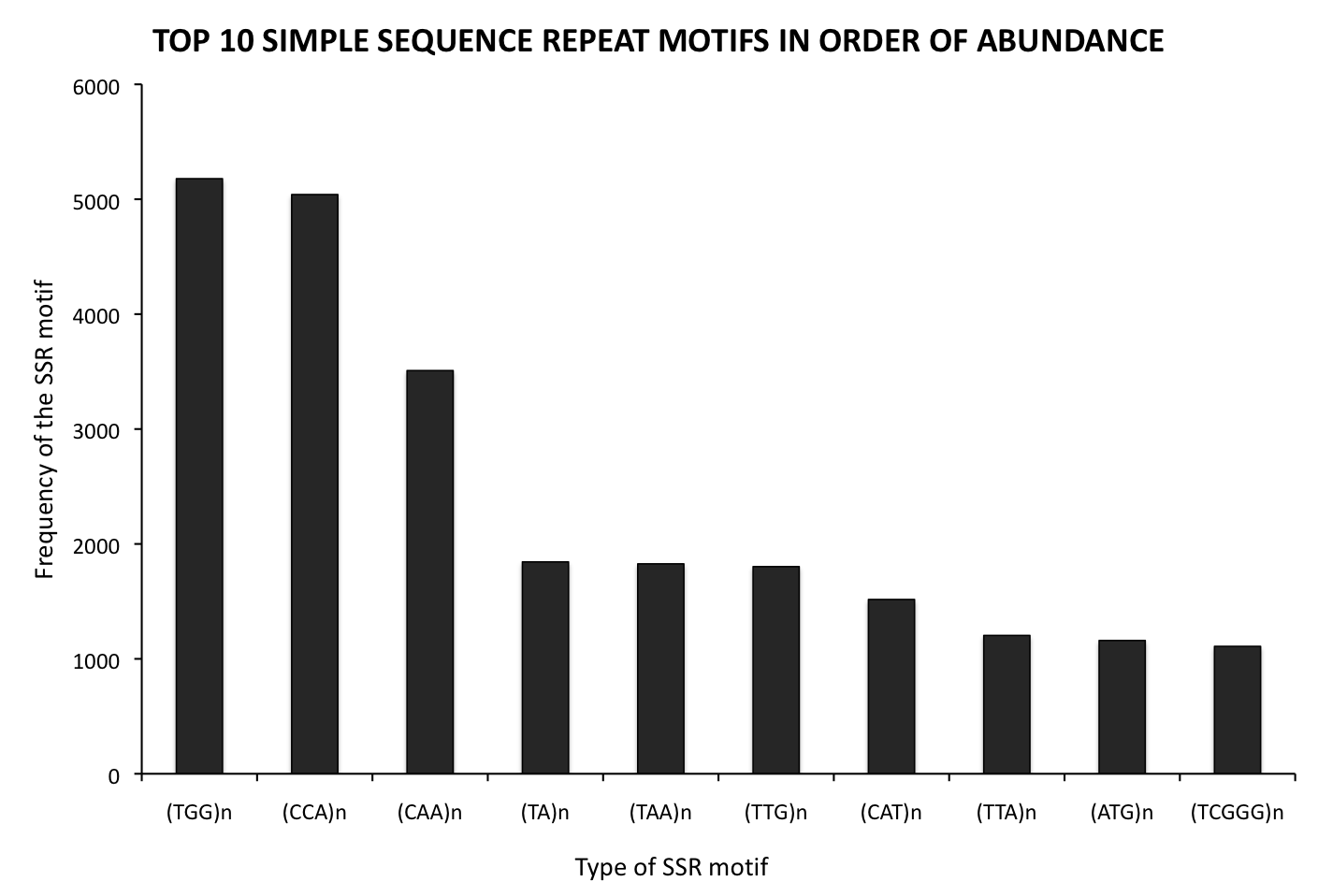


**Figure S3.** Different types of Low Complexity (LC) sequences identified in the sunflower genome expressed as percentage of total Low Complexity region.


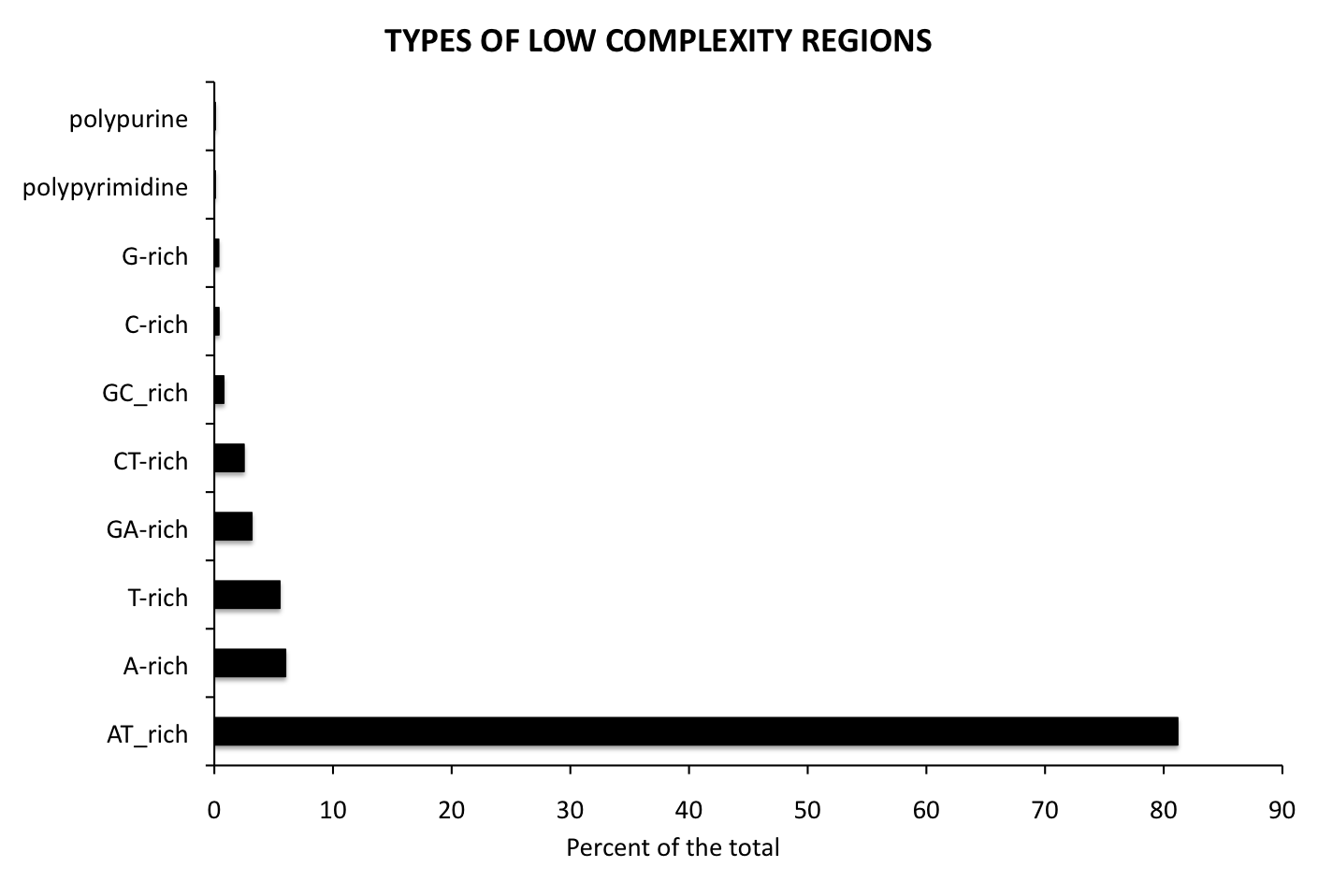


**Figure S4.** Variation in Transposable Element composition in *Arabidopsis*, Rice, Maize
and Sunflower.


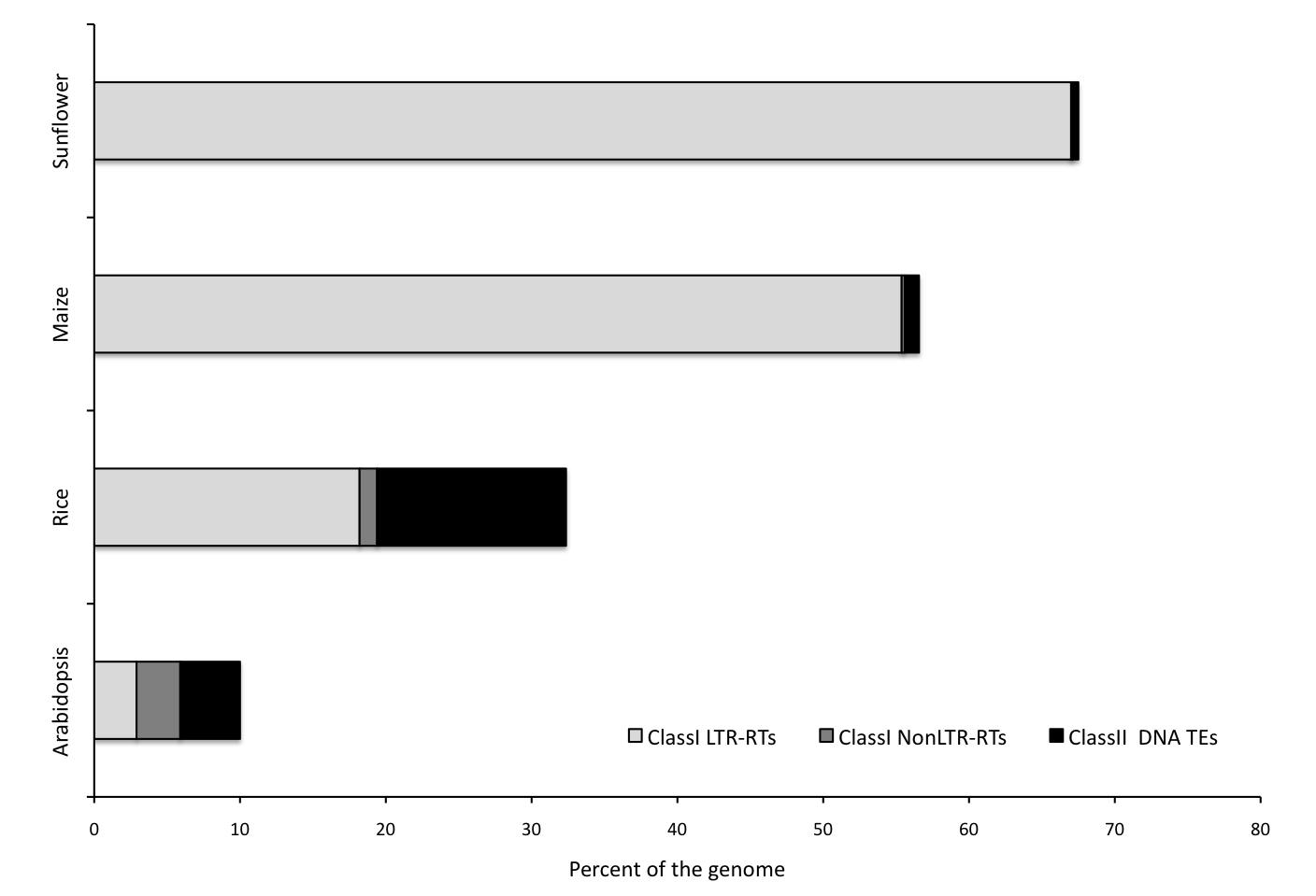


**Figure S5.** Organization of Repetitive Sequences in the BACs.


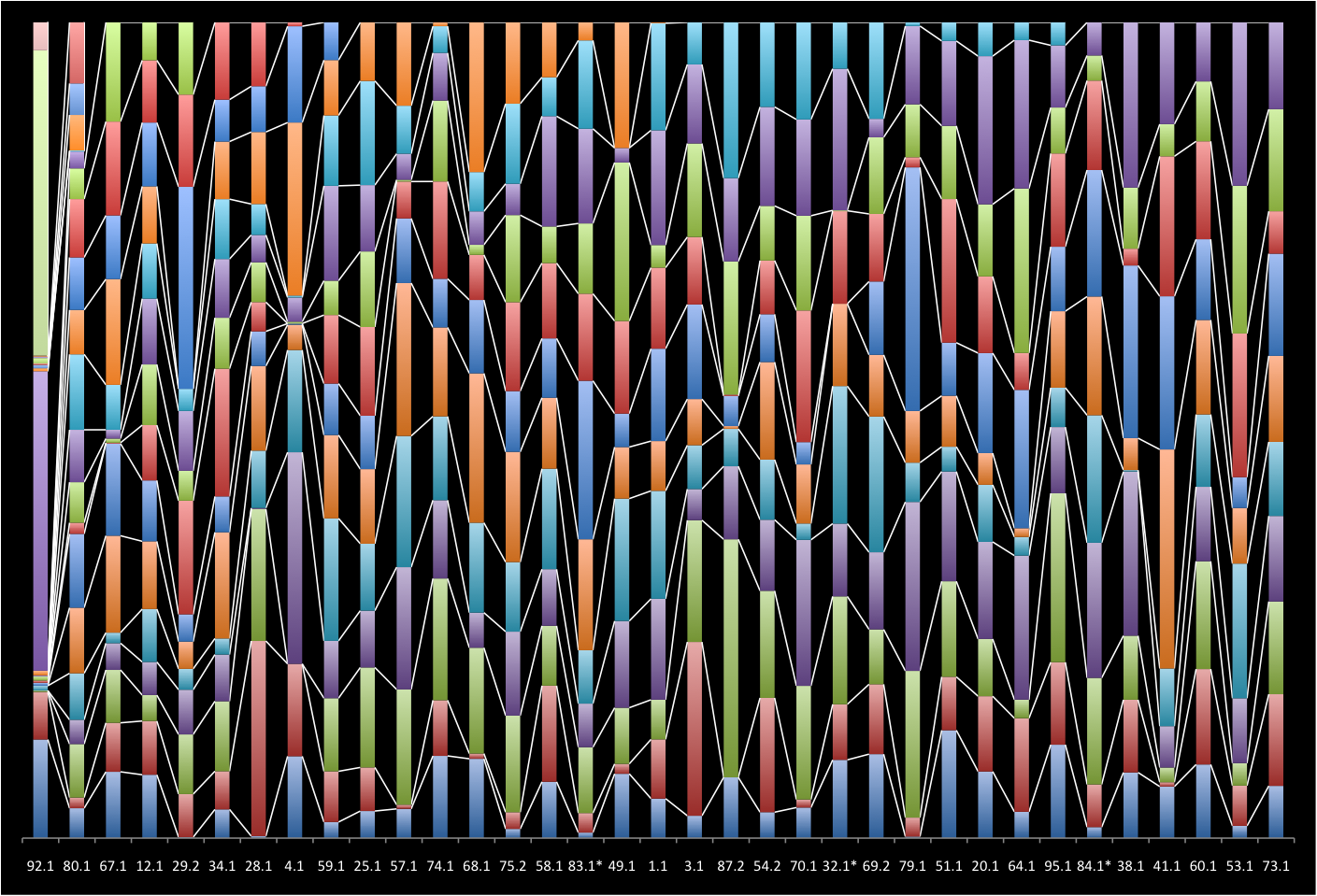


Each colored bar represents a 5kb bin and the size of the bar indicates the repetitive content within that bin. Size differences between the like colored bars indicate that there is no clear pattern of distribution of repetitive sequences across the scaffold length.

**Figure S6.** Runs non-parametric test [2] for randomness to determine the random *versus* non random distribution of repetitive sequences.


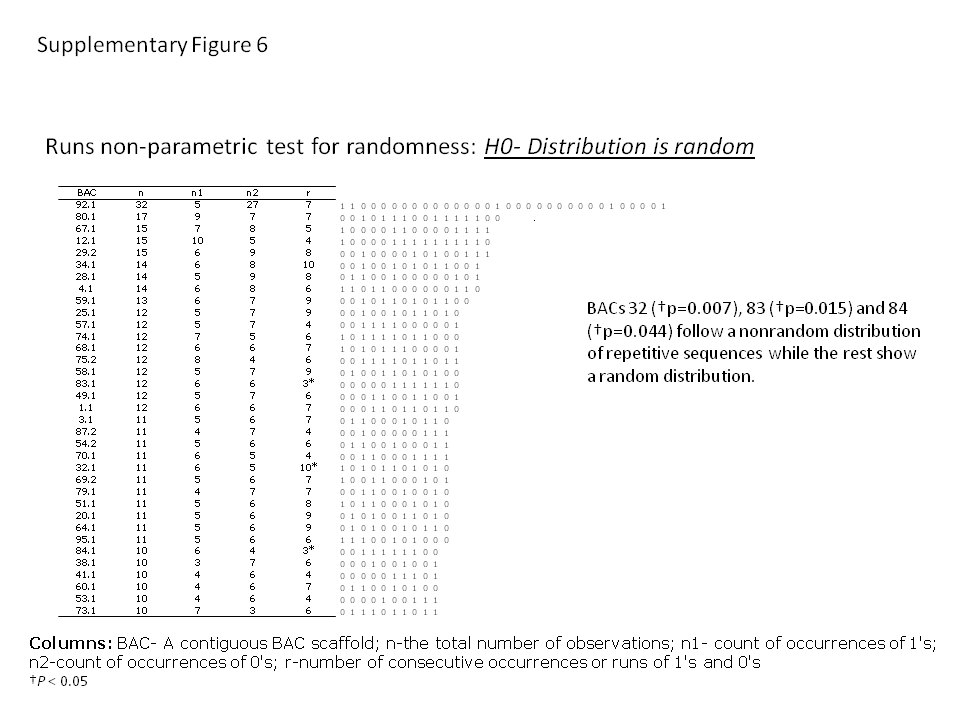


**Columns:** BAC- A contiguous BAC scaffold; n-the total number of observations; n1-count of occurrences of 1's; n2-count of occurrences of 0's; r-number of consecutive occurrences or runs of 1's and 0's † *p* < 0.05

**Figure S7.** The average divergence of 233 Transposable Element (TE) families calculated using the TE consensus approach [3–5].


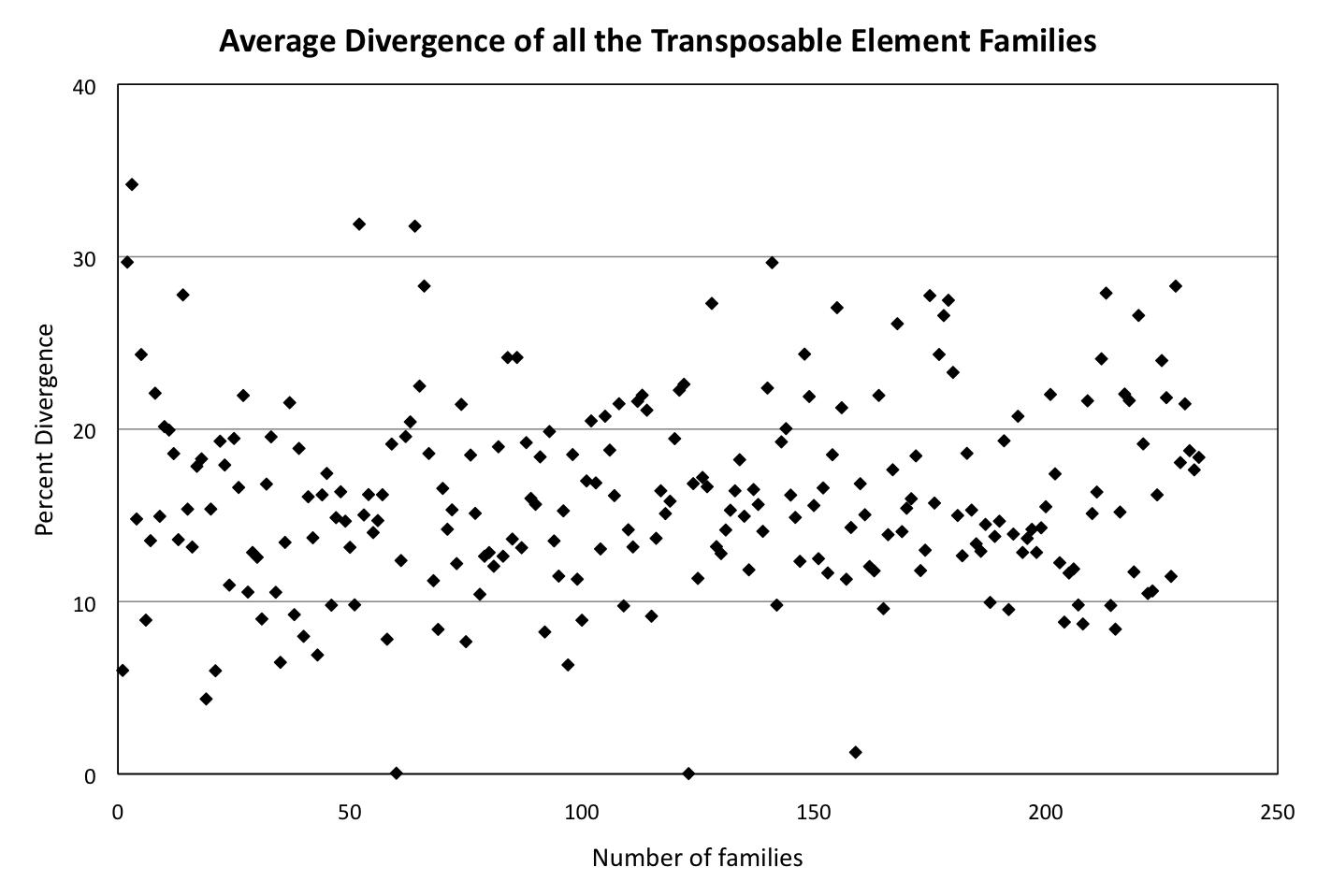


**Figure S8.** Estimation of the number and size of gene families based on the gene predictions by AUGUSTUS [6].


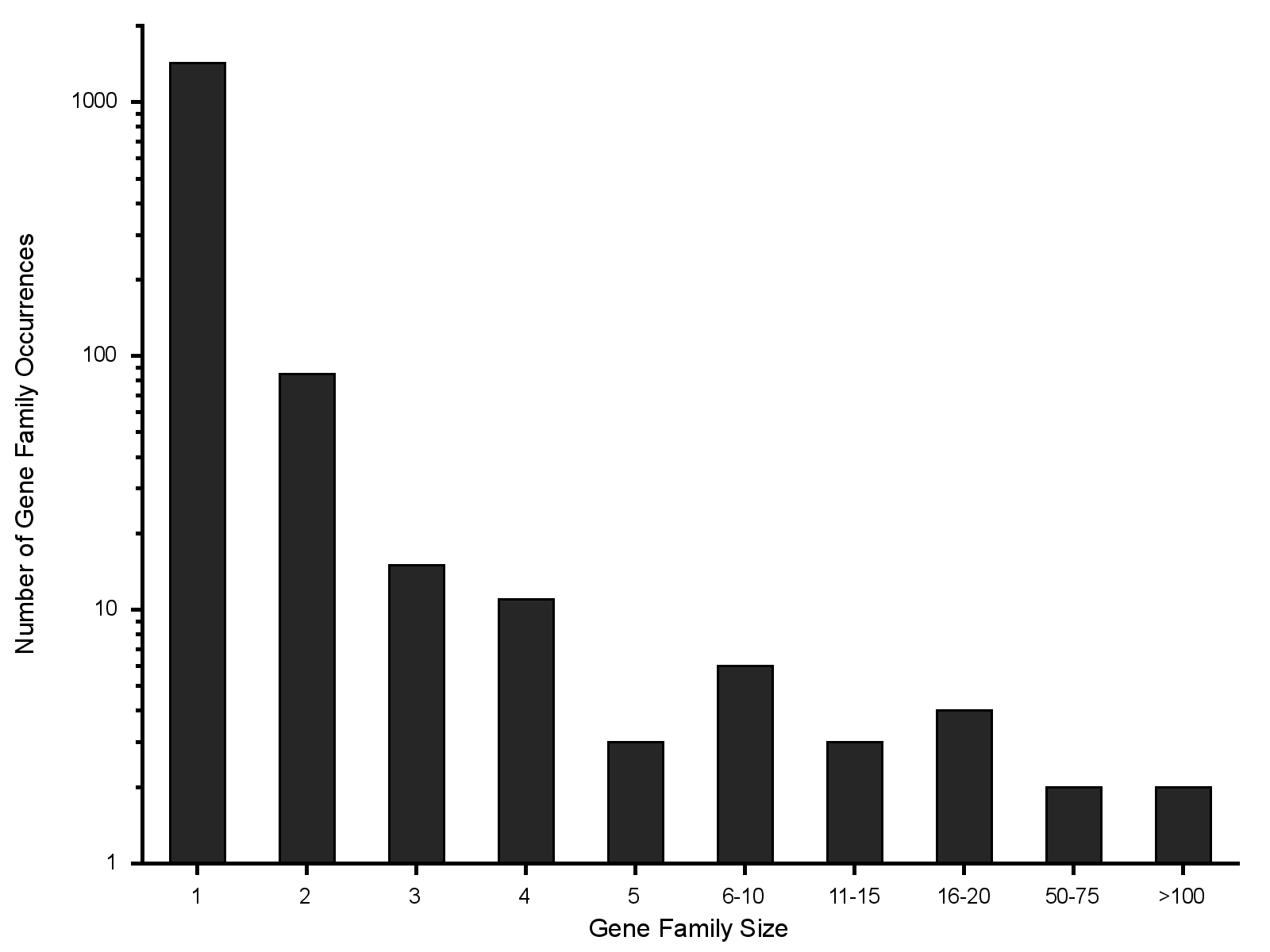


**Figure S9.** Gene Ontology (GO) annotations of the gene predictions from the repeat-masked dataset in the “Molecular Function” category using BLAST2GO (B2G) [7] using an alpha score of at least 0.6 and an ontology depth level of 3.


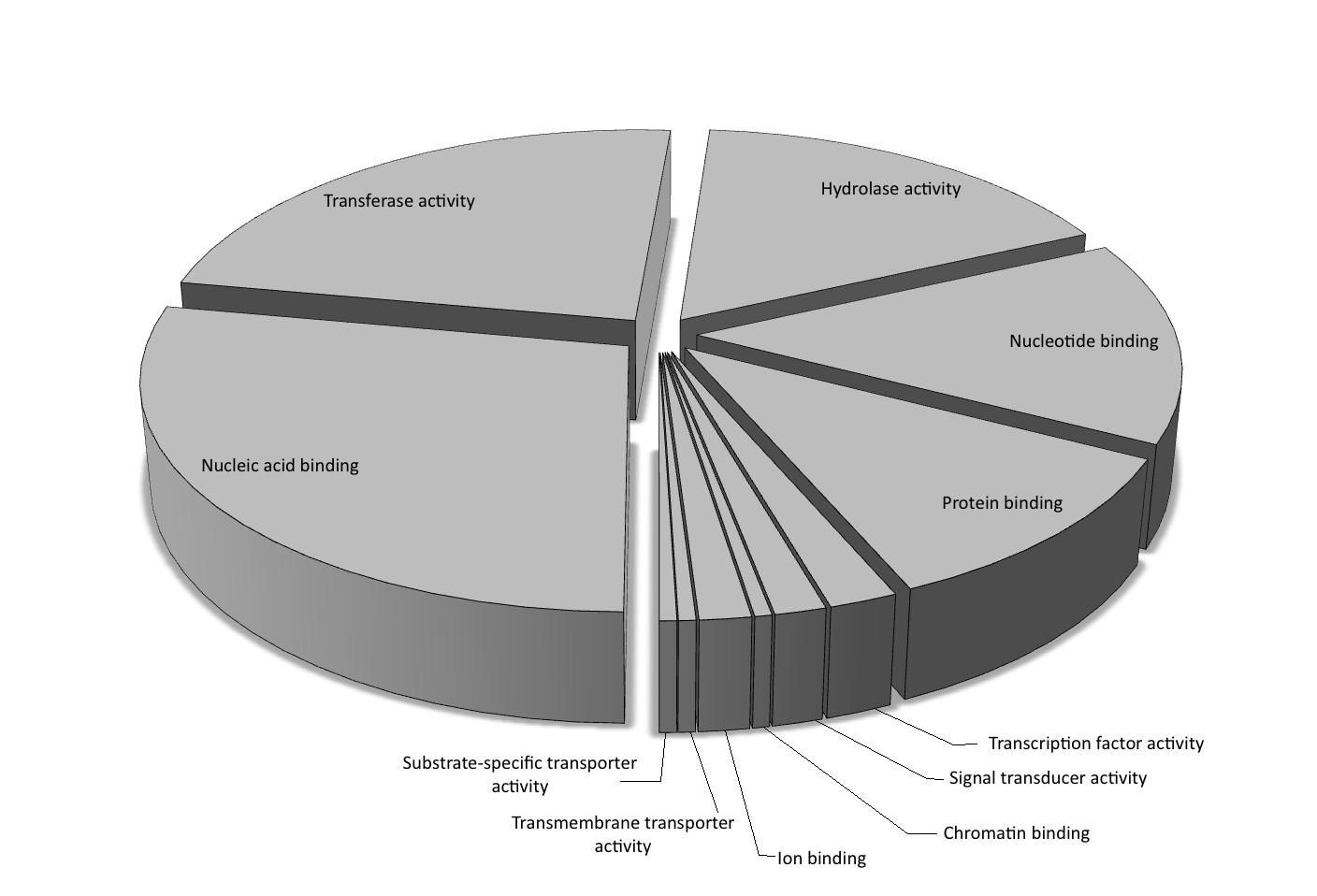


* Denotes significant difference between the masked and unmasked datasets at 99% confidence interval.

References

1. Bao, Z.; Eddy, S.R. Automated *de novo* identification of repeat sequence families in sequenced genomes. *Genome Res.* **2002**, *12*, 1269–1276.
2. Daniel, W.W. *Applied Nonparametric Statistics*; PWS Kent: Boston, MA, USA, 1990.
3. Kapitonov, V.; Jurka, J. The age of alu subfamilies. *J. Mol. Evol.* **1996**, *42*, 59–65.
4. Smit, A.F.A.; Toth, G.; Riggs, A.D.; Jurka, J. Ancestral, mammalian-wide subfamilies of line-1 repetitive sequences. *J. Mol. Biol.* **1995**, *246*, 401–417.
5. Feschotte, C.; Pritham, E.J. Computational analysis and paleogenomics of interspersed repeats
   in eukaryotes. In *Computational genomics: Current Methods*; Stojanovic, N., Ed.; Horizon Bioscience: Wymondham, Norfolk, UK, 2007; pp. 31–53.
6. Stanke, M.; Diekhans, M.; Baertsch, R.; Haussler, D. Using native and syntenically mapped cdna alignments to improve *de novo* gene finding. *Bioinformatics* **2008**, *24*, 637–644.
7. Conesa, A.; Gotz, S.; Garcia-Gomez, J.M.; Terol, J.; Talon, M.; Robles, M. Blast2go: A universal tool for annotation, visualization and analysis in functional genomics research. *Bioinformatics* **2005**, *21*, 3674–3676.

© 2014 by the authors; licensee MDPI, Basel, Switzerland. This article is an open access article distributed under the terms and conditions of the Creative Commons Attribution license (http://creativecommons.org/licenses/by/3.0/).
